# Characterization of Transstadial Transmission of *Rickettsia Amblyommatis* in *Haemaphysalis Longicornis* Using Optimized Artificial Membrane Feeding System

**DOI:** 10.4269/ajtmh.24-0782

**Published:** 2025-04-29

**Authors:** Justin Le, Molly B. Thompson, Wan-Yi Yen, Smruti Mishra, David Carlson, Andrew S. Handel, Erich R. Mackow, Jorge L. Benach, Ilia Rochlin, Hwan Keun Kim

**Affiliations:** ^1^ Center for Infectious Diseases, Stony Brook University, Stony Brook, New York;; ^2^ Department of Microbiology and Immunology, Stony Brook University, Stony Brook, New York;; ^3^ Institute for Advanced Computational Science, Stony Brook University, Stony Brook, New York;; ^4^ Department of Pediatrics, Stony Brook University, Stony Brook, New York

## Abstract

Ticks are blood-feeding arthropods and serve as vectors and reservoirs for diverse pathogens. Recent environmental changes have triggered the invasion of ticks into new geographical areas, prompting a public health alert for increased risk of tick-borne diseases. *Amblyomma* (*A.*) *americanum* (lone star tick) has emerged as the most common human-biting tick species in the eastern United States. *Amblyomma americanum* transmits multiple pathogens, including *Rickettsia* (*R.*) *amblyommatis*, the suspected cause of mild spotted fever rickettsiosis. As an invasive tick species, *Haemaphysalis* (*H.*) *longicornis* (longhorned tick) has rapidly invaded and expanded to high densities in the eastern United States. *Haemaphysalis longicornis* and *A. americanum* often share the habitat with preferential feeding on mid- to large-sized animals, such as white-tailed deer. This sympatric association of *H. longicornis* with *A. americanum* raises the potential for *H. longicornis* to acquire pathogens native to *A. americanum* during blood-feeding on the same host. In its native ranges, *H. longicornis* transmits *R. japonica* and *R. heilongjiangensis*. However, it remains unclear whether *H. longicornis* can transmit *R. amblyommatis* abundantly present in *A. americanum* in the United States. Using artificial membrane feeding, we establish that *R. amblyommatis* can stably colonize *H. longicornis* without altering feeding and molting behaviors. Transovarial transmission of *R. amblyommatis* did not occur in parthenogenetic *H. longicornis.* However, *R. amblyommatis* successfully invaded the midgut and salivary glands of *H. longicornis*, key organ tissues of rickettsial replication and horizontal transmission. Our results suggest that *H. longicornis* may serve as a vector, but not as a reservoir, for *R. amblyommatis* transmission.

## INTRODUCTION

Several environmental factors have contributed to the range and population expansion of medically important ticks, increasing tick-borne infections in the United States.^1^ Recent tick surveillance studies have demonstrated that *Haemaphysalis* (*H.*) *longicornis* Newmann, an ixodid tick native to East Asia, has rapidly invaded the eastern United States (from New York and Massachusetts in the north to Oklahoma in the west and Georgia in the south).^2^^,^^3^ Ecological modeling strongly suggests that large areas of the eastern United States provide suitable habitat for *H. longicornis*, with the expected range encompassing temperate forests of northeastern America.^4^ Locally, *H. longicornis* can become the dominant tick species in focal spots within the surveyed sites, colocalizing with native tick species of medical importance, such as *Amblyomma* (*A.*) *americanum*, *Ixodes scapularis*, and *Dermacentor* (*D.*) *variabilis*.^5^ *Haemaphysalis longicornis* can feed on diverse mammalian hosts and reproduce parthenogenetically (biological ability to produce progeny without fertilization); thus, a single female tick has the potential to create a dense population in various environmental conditions.^6^^–^^8^ In 2017, *H. longicornis* was first identified in Hunterdon County, New Jersey, and Westchester County, New York.^9^^,^^10^ However, retrospective analysis of archived tick specimens determined that *H. longicornis* was present in West Virginia as early as 2010.^11^^,^^12^ Subsequent studies determined that *H. longicornis* successfully invaded and rapidly expanded to high densities in the New York City metropolitan area and has since been documented for human bites.^7^^,^^12^^–^^15^ The rapid invasion and successful expansion of parthenogenetic human-biting tick species is unprecedented and raises a public and veterinary health alarm in the United States.^16^ Sympatric association and co-feeding on shared vertebrate hosts may provide opportunities for *H. longicornis* to acquire tick-borne pathogens from native tick species, posing a potential threat to public health as *H. longicornis* expands at a high density in local communities.^17^^–^^21^ Furthermore, host-seeking *H. longicornis* can take a partial bloodmeal from mammalian hosts infected with tick-borne pathogens and transmit them to naïve animals in the following bloodmeal in the same life stage, further enhancing the vectoral capacity of this tick species.^18^

*Amblyomma americanum* continues to expand its habitat and is found in large numbers in many parts of the United States.^22^ Importantly, *A. americanum* displays aggressive biting behavior and feeds on diverse mammalian hosts, including humans, making it a well-established vector for transmitting tick-borne pathogens and causing α-Gal allergy.^22^ Among bacterial agents, *Rickettsia* (*R.*) *amblyommatis* has been frequently identified in the surveyed ticks in several parts of the United States.^23^^–^^29^ In contrast, the current prevalence of *R. rickettsii* (causative agent of Rocky Mountain spotted fever [RMSF]) in *D. variabilis* is estimated to be less than 1%.^30^ The high prevalence of *R. amblyommatis*, combined with the aggressive biting behavior of *A. americanum*, enhances the probability of human infections with *R. amblyommatis*.^31^ Several lines of clinical and serological evidence suggest that *R. amblyommatis* is the etiological agent of mild rickettsiosis. In 2006, a partially engorged female *A. americanum* was removed from a patient who developed a macular rash at the tick bite site.^32^ A polymerase chain reaction (PCR) test confirmed the presence of *R. amblyommatis* and the absence of other tick-borne pathogens.^32^ Analysis of paired sera from patients diagnosed with probable RMSF revealed that some patients developed antibodies to *R. amblyommatis*, but not to *R. rickettsii* or *R. parkeri*.^33^^–^^35^ Those patients with specific reactivity to *R. amblyommatis* presented typical clinical manifestations of rickettsiosis with fever, headache, and myalgia.^35^ Verifying this under laboratory conditions, *R. amblyommatis* infections caused cytopathology in human endothelial cells and clinical manifestations in animal models.^36^^–^^38^

The development of an artificial membrane feeding system (AFS) for ixodid ticks significantly expanded our understanding of tick biology and the transmission of pathogenic microorganisms.^39^^–^^43^ The principal components of AFS include a temperature- and humidity-controlled feeding chamber, a receptacle with a siliconized artificial membrane, chemical and biological attractants or phagostimulants, and blood treated with chemical and biological agents. Prior AFS studies describe various experimental strategies for assembling the feeding chamber, manufacturing artificial membranes, extracting phagostimulants, and supplementing the blood with chemical and biological substances.^42^^–^^44^ The development and optimization of AFS for individual tick species allowed the investigators to study tick biology, tick transmission of pathogens, and tick–pathogen interactions.^40^^–^^44^ Previous work documented the development of semi-artificial feeding systems for *H. longicornis* with bovine or mouse skin as an artificial membrane to induce tick attachment and blood-feeding.^45^^,^^46^ However, further optimizations for AFS with silicone-based membranes have not been described for *H. longicornis*.

In this study, we describe the development and optimization of AFS for *H. longicornis* and document successful blood-feeding for all growth stages of laboratory-reared longhorned ticks. Further, we demonstrate transstadial, but not transovarial, transmission of *R. amblyommatis* strain SBU, a local isolate circulating amongst *A. americanum*, under laboratory infections of *H. longicornis*. Our results suggest that parthenogenetic *H. longicornis* may serve as a vector but not as a reservoir for *R. amblyommatis*. Our AFS model system can be used to study the limiting effects of parthenogenesis on pathogen transmission. Lastly, our work provides opportunities to use *H. longicornis* AFS as a model system to further an understanding of host–pathogen–vector interactions of invasive tick species.

## MATERIALS AND METHODS

### Cell lines and bacterial strains.

Vero cells (African green monkey kidney cells, American Type Culture Collection, Baltimore, MD) were cultured in Dulbecco’s modified Eagle’s medium (DMEM, Gibco, Chicago, IL) supplemented with 10% heat-inactivated fetal bovine serum (HI-FBS, Gibco) at 37°C in a 5% carbon dioxide atmosphere. *R. amblyommatis* strain SBU was isolated from field-collected nymphal *A. americanum* ticks. Briefly, 40 *A. americanum* nymphs were surface sterilized with 70% ethanol, rinsed with sterile phosphate buffered saline (PBS), and homogenized in 2 mL DMEM supplemented with 5% HI-FBS. *Amblyomma americanum* tick homogenates were inoculated into monolayers of Vero cells cultured with DMEM supplemented with 5% HI-FBS and 1 mM penicillin–streptomycin (10,000 U/mL, Gibco) in six-well plates. After 12 days of incubation at 34°C, two plaques were isolated and expanded on Vero cells without penicillin–streptomycin. Stocks of *R. amblyommatis* SBU were generated by differential centrifugation through 25% MD-76R solution (816 mM meglumine diatrizoate, 157 mM sodium diatrizoate hydrate, 1 mM NaH_2_PO_4_, pH 7.0; 21,000 × g, 4°C, 20 minutes) and stored at –80°C in sucrose phosphate glutamate buffer (218 mM sucrose, 3.8 mM KH_2_PO_4_, 7.2 mM K_2_HPO_4_, 4.9 mM L-glutamate, pH 7.2). Genomic and plasmid DNA samples were extracted (PureLink Genomic DNA Mini Kit, Invitrogen) and Illumina sequenced on a NexSeq 550 instrument to confirm that *R. amblyommatis* SBU (genome submission ID: SUB14880264, Genomics Core Facility, Stony Brook University, Stony Brook, NY) is closely related to *R. amblyommatis* GAT-30V (National Center for Biotechnology Information GenBank database accession no. CP003334.1) with 99.99% sequence identity.

### Tick sampling locations.

Sampling locations in Suffolk County, New York, are documented in the previous study.^5^ *Haemaphysalis longicornis* collections occurred during the spring–summer seasons (April through September) of 2022 and 2024. Questing ticks were collected from vegetation by flagging a 1 m^2^ white double-sided corduroy flag (Kaufman 8 wale) attached to a wooden pole. Ticks were brought to the laboratory and identified to species, gender, and stage under a high-power dissecting microscope using published keys.

### Artificial membrane feeding system for *H. longicornis*.

Based on our published AFS protocol for *A. americanum*,^42^ we optimized AFS for *H. longicornis* with the following modifications. Silicone membranes were prepared in a fume hood using Goldbeater’s skin (TALAS, Brooklyn, NY) with Ecoflex 00-10 Smooth-On silicone (Smooth-On, Inc, Macungie, PA) mixed with hexane (15% v/v). The single-sided tape was used to fix a sheet of Goldbeater’s skin on plastic wrap. Approximately 3 mL of the silicone mixture were then applied and spread with a small solid roller to completely cover the membrane, removing as much of the remaining silicone as possible. The membranes were cured on a flat surface for at least 24 hours. The feeding chambers were constructed with clear polycarbonate tubes (inner ring 1/1.25 inches [I.D./O.D.] and outer ring 1.25/1.5 inches [I.D./O.D.]; Plastic craft, West Nyack, NY). Using silicone sealant (Gorilla), the feeding chambers were attached to the membrane. After curing for 12 hours, the membranes were sterilized with 70% ethanol. After drying, the inner membranes were pretreated with 80 µL rabbit hair (*Oryctolagus cuniculus*, Division of Laboratory Animal Resources, Stony Brook University) extract prepared in methanol and dried for 12 hours in a fume hood.

We used laboratory-reared *H. longicornis* ticks (National Tick Research and Education Resource, Oklahoma State University, Stillwater, OK, and BEI Resources, Manassas, VA) and PCR-confirmed the absence of *Rickettsia* in DNA samples extracted from randomly selected ticks (<10 ticks per batch). All ticks were maintained at room temperature and surface-sterilized with 10% bleach for 1 minute, followed by multiple rinsing in sterile water before placement in the feeding chamber. In each feeding chamber, 15 adult ticks or 30 nymphal and three adult ticks were placed close to the membrane with a 3 cm layer of rabbit hair on top. For larval feeding, *H. longicornis* egg clutches were loaded into syringes with wide-bore openings and kept in the incubator. After hatching, *H. longicornis* larvae were briefly chilled on ice and gently pushed into the feeding chamber. A white sponge was placed close to the artificial membrane to restrict larval tick movement. Modified plastic stoppers (WW-12X, Caplugs Inc, Buffalo, NY) with a central hole and mosquito netting were placed to close individual feeding receptacles. The feeding chambers were placed into the six-well plate, with each well containing 4 mL of the complete sheep blood (defibrinated sheep blood [Hemostat Laboratories, Dixon, CA] supplemented with 1 M glucose, 4 mM 0.1% adenosine triphosphate, 1 mM amphotericin B [Gibco], and 1 mM penicillin–streptomycin [10,000 U/mL, Gibco]). The assembled units were placed in a 2.5-gallon aquarium tank (Aqueon, All Glass Aquarium AAG10002 Tank) filled with sterile water (up to 0.5 inches above the bottom of the tank), with a temperature probe and hygrometer secured to the tank. Two reptile heating pads (6W, 7 × 4 inches, Wedoelsim) were placed at the bottom of the tank to create differential heating (water temperature at 37°C and relative humidity above 85%) to facilitate downward feeding behavior toward the silicone membrane. The aquarium tanks were placed in an incubator at 25°C, with a photoperiod of 16:8 hours (light:dark) using an aquarium light-emitting diode lamp (AQQA aquarium light) connected to a timer (GE 24-hour heavy-duty indoor plug-in mechanical timer). Blood in the feeding chambers was replaced every 12 hours until repletion (up to 5 days or 9 days post-feeding for nymphal and adult ticks, respectively). For blood changes, the feeding chambers were removed from the six-well plate and rinsed with preheated sterile PBS. After washing, the feeding chambers were placed in freshly prepared six-well plates containing pre-warmed complete sheep blood. For *R. amblyommatis* infections, *H. longicornis* ticks were exposed to the complete sheep blood spiked with 2–16 × 10^6^ plaque forming unit *R. amblyommatis* SBU, starting at 36 hours post-feeding for the next six blood exchanges (a total of 72 hours). Mock infection units received the same complete sheep blood lacking *R. amblyommatis* SBU. Throughout the experiments, we counted unfed, partially fed, repleted, and dead ticks and kept them separately in a desiccator with an oversaturated potassium chloride solution to maintain approximately 85% relative humidity at room temperature. Engorged female *H. longicornis* ticks completed oviposition and produced eggs, which were counted for pools of 50 eggs and subjected to PCR analysis. Some eggs were allowed to hatch into larvae, which were subjected to PCR analysis in pools of 20.

### DNA extraction and PCR analysis for *R. amblyommatis*.

*Haemaphysalis longicornis* ticks were disinfected by washing with 10% bleach and sterile PBS. Tick samples were placed in 2.0 mL screw cap microcentrifuge tubes containing approximately 20–25 0.1 mm zirconia beads, 2–3 2.3 mm zirconia beads (Biospec), and 300 µL DNAzol (DN127, Molecular Research Center, Cincinnati, OH). Tick samples were homogenized using Mini-Beadbeater 16 (Biospec, Bartlesville, OK) for 90 seconds and visually checked for complete homogenization. Tissue fragments were pelleted by centrifugation at 10,000 × g for 10 minutes at room temperature. For DNA precipitation, 250 µL supernatant was mixed with 125 µL ethanol and centrifuged at 5,000 × g for 10 minutes. The pellet was washed twice with 200 µL 75% ethanol, resuspended in 50 µL sterile water, and stored at 4°C for PCR analysis.

We used two primers (RambFWD: 5′-TTCCTGTAAATAAATGCAAGCCTCT-3′, RambREV: 5′-ATGGCAGTCAACATTACCAAAGC-3′, synthesized by Integrated DNA Technologies, Research Triangle Park, NC) defining a 157 base pair (bp) region specific for *R. amblyommatis*. PCR reactions (50 µL) contained 100–300 ng template DNA, 2 U of Z-Taq polymerase (Takara Bio, San Jose, CA), 1 × Z-Taq buffer containing 3 mM Mg^2+^, 1 × PCR enhancer (Invitrogen), 0.2 µmol of each primer, and 200 µmol dNTP mix. The following thermocycling condition was used to amplify the target region on *R. amblyommatis* chromosomal DNA: 2 minutes at 98°C, 25 cycles of 5 seconds at 98°C, 10 seconds at 53°C, and 5 seconds at 72°C, followed by 2 minutes at 72°C. Genomic DNA was prepared from Renografin-purified *R. amblyommatis* and used as a positive control (PureLink Genomic DNA Mini Kit, Invitrogen). After PCR, each sample was analyzed by 1.0% Tris-Acetate EDTA agarose gel electrophoresis with 100 bp DNA markers (New England Biolabs, Ipswich, MA) and stained with GelRed Nucleic Acid Stain [Sigma]). Positive PCR fragments were excised, and Sanger sequenced (DNA Sequencing Facility, Stony Brook University) to confirm the presence of *R. amblyommatis*-specific sequences.

### Immunofluorescence microscopy.

Disinfected ticks were dissected using entomological forceps.^47^ After separating the scutum from the body, salivary glands and midgut tissues were isolated and cleaned using sterile PBS. Tick organ tissues were mounted on microscope slides pretreated with poly-D-lysine (Gibco). Tissue samples were fixed in 4% paraformaldehyde for 30 minutes at room temperature. Then, the samples were incubated in 0.1% Triton X-100 for 15 minutes at room temperature before blocking them with 1% bovine serum albumin for 60 minutes at room temperature. To determine the presence of *R. amblyommatis*, the tissue samples were incubated with rabbit α-*R. conorii* antiserum (1:500 in PBS, cross-reactive to *R. amblyommatis*) at 4°C for 18 hours. The microscope slides were washed three times in PBS for 5 minutes in each incubation. Next, the samples were incubated with goat α-rabbit Alexa Fluor 488 (1:400 in PBS, Jackson ImmunoResearch Laboratories) and ActinRed (1:200 in PBS, Invitrogen) in the dark for 3 hours at room temperature. After this incubation, NucBlue Live ReadyProbes Reagent (Invitrogen) was incubated with the slides for 30 minutes. After washing the slides in PBS, ProLong Diamond Antifade Mountant (Invitrogen) was used as the mounting media, with a coverslip covering the tissue on the slide. Microscopic images were obtained with Zeiss LSM 980 with Airyscan 2 (Central Microscopy Imaging Center, Stony Brook University).

### Biosafety and biosecurity.

Research was performed in accordance with institutional guidelines following experimental protocol review, approval, and supervision by the Institutional Biosafety Committee at Stony Brook University. Experiments with field-collected *A. americanum* lysates were performed in biosafety level three containment.

## STATISTICAL ANALYSES

Student’s *t*-test was performed to analyze the statistical significance of tick body weights (Prism, GraphPad).

## RESULTS

### AFS optimization for *H. longicornis*.

**Figure 1.**
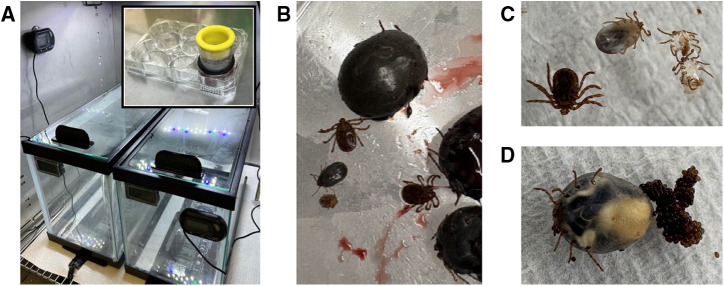
The artificial membrane feeding system supports the blood-feeding of *Haemaphysalis* (*H.*) *longicornis* ticks in all growth stages. (**A**) Photographic images of the assembled six-well plate (inset) and feeding chamber. Representative images of (**B**) adults and nymphs (repleted versus non-fed) recovered from the feeding experiments, (**C**) an engorged nymph and a freshly molted adult with exuviae, and (**D**) a fully engorged adult with eggs.

Based on the optimized AFS protocol for *A. americanum*, we performed experiments to optimize experimental conditions for laboratory-reared *H. longicornis*.^42^ Our results suggested that the application of silicone-based Goldbeater’s skin as an artificial membrane with rabbit hair extract and physical constraints toward the membrane facilitated consistent *H. longicornis* attachment and feeding. Further, differential heating promoted the tick attachment and feeding behavior of *H. longicornis* on an artificial membrane (Figure 1).

### Transstadial transmission of *R. amblyommatis* in *H. longicornis*.

**Figure 2.**
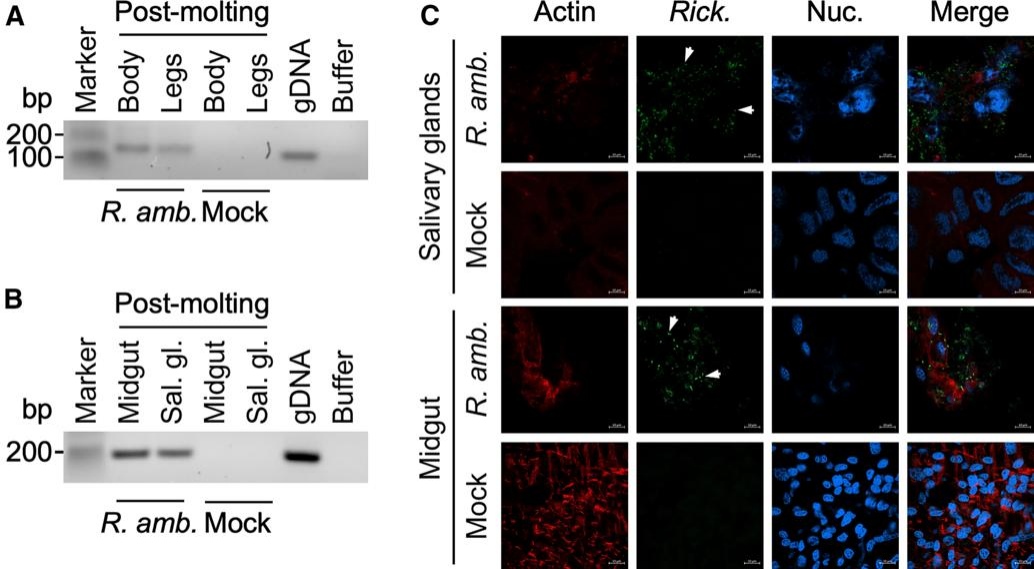
*Rickettsia* (*R.*) *amblyommatis* is transstadially transmitted from nymphs and colonizes the salivary glands and midgut of *Haemaphysalis* (*H.*) *longicornis* adults. Cohorts of *H. longicornis* nymphs ingested sheep blood supplemented with *R. amblyommatis* or SPG buffer (Mock) through the artificial membrane feeding system. After repletion, engorged nymphal ticks molted into adults. Freshly molted adult ticks were examined to determine the presence of *R. amblyommatis* in organ tissues by (**A** and **B**) polymerase chain reaction (PCR) or (**C**) immunofluorescent microscopic analyses. Red = actin; green = *Rickettsia* (white arrows); blue = nucleus.

Using the optimized AFS for *H. longicornis*, we performed three independent experiments with *H. longicornis* nymphs (30 ticks per experiment) to determine the vertical transmission of *R. amblyommatis* (Table 1). *Haemaphysalis longicornis* nymphs exhibited consistent and comparable attachment and feeding rates between mock (42% total with 4% partially fed and 38% repleted ticks) and *R. amblyommatis* infected (42% total with 8% partially fed and 34% repleted ticks) groups (Table 1). Despite our best efforts to minimize environmental contamination (surface sterilization of the incubators and feeding chambers, use of fresh sterile water and blood, and the addition of antibiotics and antifungal agents), we continued to encounter fungal contamination of our feeding chamber, which affected the survival of *H. longicornis* ticks attached to the artificial membrane (18–21% dead ticks). Despite these technical challenges, most repleted nymphs fed comparable amounts of blood (*P* >0.05) and became adults (88–90% for mock and *R. amblyommatis*-infected groups, Table 2). Among those fed with blood spiked with *R. amblyommatis*, PCR analysis determined the presence of *R. amblyommatis* in the legs and body of all molted adults, suggesting a systemic colonization with *R. amblyommatis* (Figure 2A). Corroborating this finding, PCR and immunofluorescence microscopic analyses (IFA) identified coccobacillary *R. amblyommatis* in salivary glands and midguts of *R. amblyommatis*-infected ticks (Figure 2B and C). In contrast, the PCR and IFA analyses failed to detect *R. amblyommatis* in mock-infected ticks (Figure 2).

**Table 1:** Artificial membrane feeding studies with nymphal and adult *Haemaphysalis longicornis* ticks

Groups*	Exps^†^	Nymphs^‡^					Exps^†^	Adults^‡^				
		U	P	R	D	T		U	P	R	D	T
*Rickettsia amblyommatis*	3	36 (40%)	7 (8%)	31 (34%)	16 (18%)	90 (100%)	5	29 (39%)	12 (16%)	21 (28%)	13 (17%)	75 (100%)
Mock	3	33 (37%)	4 (4%)	34 (38%)	19 (21%)	90 (100%)	5	32 (43%)	3 (4%)	16 (21%)	24 (32%)	75 (100%)

D = dead; Exps = experiments; P = partially fed; R = repleted; T = total; U = unfed.

* Two experimental groups.

^†^ Number of independent experiments.

^‡^ Number of ticks and relative abundance as percentages in parenthesis.

**Table 2:** Molting efficiencies of nymphal *Haemaphysalis longicornis* ticks fed on artificial membrane feeding system

Groups*	Exps^†^	Nymphs^‡^		
		Repleted	Molted	Weight^§^
*Rickettsia amblyommatis*	3	31	27 (90%)	4.34 ± 0.98
Mock	3	34	24 (88%)	4.42 ± 0.80

Exps = experiments.

* Two experimental groups.

^†^ Number of independent experiments.

^‡^ Number of ticks and relative abundance as percentages in parenthesis.

^§^ Average weight (±SD) of engorged ticks in milligrams.

### Transovarial transmission of *R. amblyommatis* in *H. longicornis*.

**Figure 3.**
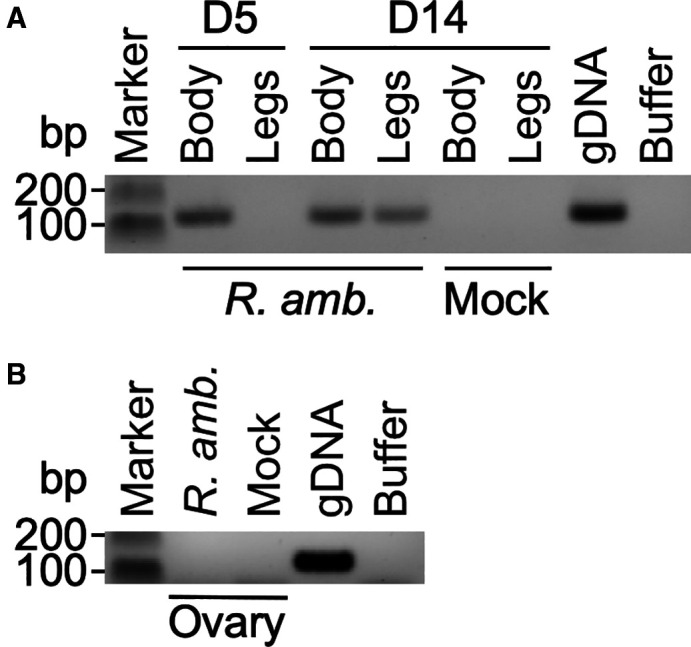
*Rickettsia* (*R.*) *amblyommatis* infects distal organ tissues but fails to colonize ovaries in adult *Haemaphysalis* (*H.*) *longicornis*. (**A**) Engorged *H. longicornis* ticks were dissected and polymerase chain reaction (PCR)-tested for *R. amblyommatis* on day 5 or 14, post-feeding. (**B**) Furthermore, ovaries from adult ticks were examined for *R. amblyommatis* by PCR.

To determine the transovarial transmission efficiency of *R. amblyommatis* in *H. longicornis* ticks, we performed five independent AFS studies with freshly reared *H. longicornis* adults (devoid of *R. amblyommatis* infections, 15 ticks per experiment, Table 1). Similar to *H. longicornis* nymphs, adult ticks demonstrated consistent and comparable attachment and feeding behavior (unfed ticks ranging between 39% [*R. amblyommatis*] and 43% [Mock]). However, we observed a slight increase in the number of dead adult ticks in the mock-infected group (32%) compared with *R. amblyommatis*-infected ticks (17%). *R. amblyommatis*-specific PCR analysis identified the presence of *R. amblyommatis* in the bodies of repleted ticks on 5- and 14-days post-feeding (dpf, Figure 3A). Interestingly, the same PCR analysis failed to detect *R. amblyommatis* in the legs of repleted adults on 5-dpf, suggesting that most *R. amblyommatis* had not disseminated. However, by 14-dpf, *R. amblyommatis* was present in the legs of repleted adults, suggesting a fully disseminated infection. Both groups of repleted ticks (Mock and *R. amblyommatis*) ingested comparable amounts of blood with successful oviposition, producing more than 1,000 eggs. However, all egg samples were PCR-negative for *R. amblyommatis* (1,300 eggs in *R. amblyommatis*-infected and 600 eggs in Mock-infected groups). We performed *R. amblyommatis*-specific PCR analysis with ovaries isolated from engorged *H. longicornis* adults to assess whether *R. amblyommatis* invades ovaries during vitellogenesis. However, ovaries were uniformly negative for *R. amblyommatis* (Figure 3B). To confirm the absence of transovarial transmission of *R. amblyommatis* in *H. longicornis*, some eggs were separated and left to hatch into larvae. Corroborating our PCR analysis of egg and ovary samples, PCR testing of larvae failed to detect the presence of *R. amblyommatis* (1,300 larvae in *R. amblyommatis*-infected and 1,000 larvae in Mock-infected groups).

### AFS infection of *H. longicornis* larvae with *R. amblyommatis*.

**Figure 4.**
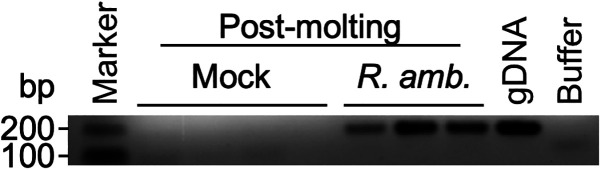
*Rickettsia* (*R.*) *amblyommatis* is transstadially transmitted from larvae and persists in *Haemaphysalis* (*H.*) *longicornis* nymphs. *H. longicornis* larvae were placed in the artificial membrane feeding system (AFS) to feed sheep blood supplemented with *R. amblyommatis* or SPG buffer (Mock). After repletion, engorged larval ticks molted into nymphs. Freshly molted nymphal *H. longicornis* ticks were polymerase chain reaction (PCR)-tested for *R. amblyommatis*.

The small body size and short hypostome of larval ticks pose significant challenges in facilitating blood-feeding through AFS. Because the optimized AFS for *H. longicornis* supported the blood-feeding of nymphal and adult ticks, we tested its capacity to support the blood-feeding of larval *H. longicornis* ticks. To avoid technical difficulties working with active larvae, we placed egg clutches in open-bore syringes with cotton caps and incubated them in the incubator. After hatching into larvae, we ice-shocked larval ticks, gently pushed transiently immobilized ticks into the feeding chamber, and placed a white sponge on top to promote their movement toward the artificial membrane. After 5 days of feeding, we observed successful engorgement of mock (135 out of 632 larvae, 21% repleted) and *R. amblyommatis*-infected (93 out of 736 larvae, 13% repleted) groups. All repleted larval ticks molted into nymphs without noticeable differences. PCR analysis confirmed the presence of *R. amblyommatis* in the infection group (Figure 4).

### Field-collected *H. longicornis* nymphs and adults are not infected with *R. amblyommatis*.

Our recent tick survey in 2022 documented that *H. longicornis* has successfully invaded and expanded in the northern Appalachian Forest ecological zones on Long Island.^5^ When revisited in 2024, we noticed abundant *H. longicornis* populations at the same collection sites, confirming their persistent and continuing expansion on Long Island. When examined by PCR, we failed to detect *R. amblyommatis* in field-collected nymphal (520 nymphs in 2022 and 1,220 nymphs in 2024) and adult *H. longicornis* ticks (170 adults in 2022 and 200 adults in 2024). Our results suggest that, despite successful *R. amblyommatis* colonization and transstadial transmission in *H. longicornis* under laboratory conditions, spillover has not occurred between *A. americanum* and *H. longicornis* in the field.

## DISCUSSION

*Haemaphysalis longicornis* serves as a vector and reservoir for a wide range of viral, bacterial, and parasitic organisms of human and veterinary concerns in its native and invasive ranges (e.g., New Zealand and Australia).^48^^–^^53^ In addition, *H. longicornis* bites are positively associated with α-Gal allergy, in which patients elicit delayed allergic responses through immunoglobulin E recognizing galactose-α-1,3-galactose in red meat in Japan.^54^ However, it remains unclear whether *H. longicornis* contributes to tick-borne pathogen transmission in the United States, except for bovine infectious anemia caused by *Theileria* (*T.*) *orientalis* (Ikeda strain). A series of studies confirmed that *H. longicornis* ticks in Virginia were PCR-tested positive for *T. orientalis* and served as a vector for *T. orientalis* outbreak in a cattle farm.^55^^–^^57^ Moreover, a recent tick survey and molecular testing determined that *H. longicornis* ticks in Virginia carry Bourbon virus, a tick-borne RNA virus first discovered in Bourbon County, Kansas, in 2014.^20^ In another study, investigators demonstrated that laboratory-reared *H. longicornis* ticks infected with Heartland bandavirus, a causative agent of tick-transmitted febrile illness with thrombocytopenia and leukopenia, transmitted the virus to offspring and caused seroconversion of infected BALB/c mice after blood-feeding.^58^ Similarly, *H. longicornis* exhibited vector competence for the Powassan virus, a North American tick-borne encephalitis flavivirus, under laboratory conditions.^59^ In contrast, when assessed for the ability to serve as a vector for *Borrelia burgdorferi*, laboratory-reared *H. longicornis* ticks failed to pass the Lyme spirochetes to the next life stage, corroborating a low infection rate (one out of 668 ticks) observed in a recent tick survey in Pennsylvania.^18^^,^^60^ Similarly, another study demonstrated that laboratory-reared *H. longicornis* could not serve as a vector for *Anaplasma phagocytophilum* Ap-ha, a variant pathogenic for humans, dogs, and horses, by demonstrating that *H. longicornis* ticks acquired Ap-ha during blood-feeding on infected goats but failed to maintain or transmit the pathogen through developmental stages or to a naïve goat during blood-feeding.^61^ A separate tick survey found that one adult female and one nymphal tick (out of 152 adults and 113 nymphs) were infected with *Anaplasma phagocytophilum* Ap-ha in Pennsylvania.^17^ Of note, a previous study reported that laboratory-reared *H. longicornis* acquired *R. rickettsii* from infected guinea pigs and transmitted the organism to naïve animals between developmental stages.^62^ Although *H. longicornis* is an established vector for *R. japonica* in its native range, it remains unknown whether invasive *H. longicornis* can acquire, maintain, or transmit other *Rickettsia* species prevalent in ticks native to the United States.

The rapid invasion of human-biting *H. longicornis* into new geographical areas, and its increasing local abundance in the eastern United States raises significant public and veterinary health concerns. Unlike native ixodid tick species, a single-engorged female *H. longicornis* can infest the local environment and rapidly increase its population. This invasive tick species has been known to vector multiple pathogens, such as severe fever with thrombocytopenia syndrome virus, Nairobi sheep disease virus, *Rickettsia* species (spp.)., *Anaplasma* spp., *Ehrlichia* spp., and *Theileria* spp. in East Asia.^48^^–^^53^ Thus far, *T. orientalis* is the only documented pathogen transmitted by *H. longicornis* in the United States.^55^^–^^57^ Similar transmission records of *T. orientalis* by invasive *H. longicornis* have been reported in Australia and New Zealand.^63^^,^^64^ *H. longicornis* ticks have been found on diverse mammalian species, such as opossums (*Didelphis virginiana*), domestic dogs (*Canis lupus familiaris*), cottontail rabbits (*Sylvilagus* spp.), white-footed mice (*Peromyscus leucopus*), and white-tailed deer (*Odocoileus virginianus*), some of which are often colonized with native tick species in the United States.^19^^,^^21^ Thus, active and passive tick surveillance efforts must be continued to document any pathogen spillovers in *H. longicornis* in the United States.

Prior studies determined that *H. longicornis* ticks are infected with SFG rickettsiae, predominantly with *R. heilongjiangensis* (Far-Eastern spotted fever) or *R. japonica* (Japanese spotted fever) in East Asia.^53^^,^^65^^,^^66^ Geographic overlap with native ticks (e.g., *D. variabilis* [a vector for *R. rickettsii*], *A. maculatum* [a vector for *R. parkeri*], and *A. americanum* [a tick species frequently infected with *R. amblyommatis*]) and shared mammalian hosts increase the possibility of *H. longicornis* exposure to rickettsial organisms prevalent in the United States. As reported by others and described in this study, tick surveys have not reported the presence of *R. rickettsii*, *R. parkeri*, and *R. amblyommatis* in field-collected *H. longicornis* in the United States.^67^ Under laboratory conditions, investigators demonstrated that *H. longicornis* can maintain *R. rickettsii* between generations at a reduced capacity compared with native tick species, such as *D. variabilis* and *A. americanum*.^62^ In a recent study conducted in South Korea, high-throughput sequencing analyses of 16S ribosomal RNA identified *R. rickettsii* in field-collected *H. longicornis* nymphs and adults. Given that *R. rickettsii* infection rates in native tick populations have remained low, an extensive tick survey may uncover the presence of *R. rickettsii* in invasive *H. longicornis* ticks in the United States. On the other hand, the infection rates of two other rickettsial pathogens, *R. parkeri* and *R. amblyommatis*, in native tick species are much higher, posing an increased risk of pathogen spillovers through co-feeding on a shared mammalian host.

In this study, we assessed the susceptibility of *H. longicornis* to *R. amblyommatis* SBU, an isolate obtained from *A. americanum* in Long Island, an insular environment where *H. longicornis* ticks have rapidly expanded, sharing habitats and vertebrate hosts with *A. americanum*. Both larval and nymphal *H. longicornis* ticks acquired *R. amblyommatis* by feeding on the artificial membrane and maintained the pathogen into the next growth stages. Notably, both experimental groups (*R. amblyommatis* versus Mock) exhibited comparable survival and molting rates, indicating that *R. amblyommatis* infections did not perturb *H. longicornis* feeding and molting processes. Despite successful oviposition and egg production of artificially fed *H. longicornis* adults, ovaries, eggs, and larval ticks remained free of *R. amblyommatis*. A previous study documented an inefficient transovarial transmission of *R. rickettsii* in *H. longicornis* fed on New Zealand white rabbits.^62^ The transovarial transmission efficiencies for *R. heilongjiangensis* or *R. japonica* have not been examined under laboratory conditions. Recent studies suggest that *Coxiella*-like endosymbiont stably infects *H. longicornis* and has the potential to modulate *H. longicornis* development, vitellogenesis, and oogenesis, which may impact the fitness of *H. longicornis* to *Rickettsia* spp. exhibiting varying degrees of virulence (from most virulent to least virulent, *R. rickettsii* > *R. japonica* > *R. amblyommatis*).^68^^,^^69^ The inability of *H. longicornis* to maintain *R. amblyommatis* through transovarial transmission, even under laboratory conditions, suggests that *H. longicornis* may have limited capacity to serve as a reservoir for *R. amblyommatis*.

Despite recent AFS developments for diverse tick species, an optimized AFS for *H. longicornis* has not been described.^39^ Prior studies reported AFS applications for *H. longicornis* using mouse skins as a source for artificial membranes.^45^^,^^46^ Both studies successfully used a semi-artificial mouse skin membrane feeding system to characterize tick–parasite interactions. However, these methods were technically challenging and relied on laboratory animals, limiting their broader adoption and application. In the present study, we optimized a silicone membrane-based AFS for *H. longicornis* to facilitate efficient attachment and blood-feeding. Using the optimized AFS, we demonstrated successful repletion of *H. longicornis* in all growth stages and subsequent molting into the next growth stages. In addition, the parthenogenetic nature of *H. longicornis* enables the investigators to use this tick organism as an excellent model system to study the transovarial transmission of tick-borne pathogens.

## CONCLUSION

Overall, our study highlights the utility of an optimized silicone membrane-based AFS for *H. longicornis*. It provides a robust platform for investigating the biology of this invasive tick species and its role in pathogen transmission. Our findings contribute to a growing body of knowledge on the interaction between *H. longicornis* and *Rickettsia* spp., using a system that can be expanded to explore the transmission of other tick-borne pathogens with veterinary and public health significance.

## Data Availability

The data and unique materials that support the findings of this study are available from the corresponding authors upon request.
